# Subcritical Water Extraction of Phenolic Compounds from Onion Skin Wastes (*Allium* *cepa* cv. Horcal): Effect of Temperature and Solvent Properties

**DOI:** 10.3390/antiox9121233

**Published:** 2020-12-04

**Authors:** Óscar Benito-Román, Beatriz Blanco, María Teresa Sanz, Sagrario Beltrán

**Affiliations:** Department of Biotechnology and Food Science (Chemical Engineering Section), University of Burgos, 09001 Burgos, Spain; bblanco@ubu.es (B.B.); tersanz@ubu.es (M.T.S.); beltran@ubu.es (S.B.)

**Keywords:** onion, extraction, subcritical water, quercetin, polarity, valorization

## Abstract

The valorization of onion skin wastes (OSW) through the extraction, identification, and quantification of phenolic compounds was studied in this work, using subcritical water in a semicontinuous extractor (2.5 mL/min; 105–180 °C; 5 MPa). The extraction of flavonoids resulted to be fast (<30 min) and temperature sensitive (maximum at 145 °C; total flavonoids, 27.4 ± 0.9 mg/g dry OSW (DOSW)). The experimental results were fitted to the Weibull model. The influence of the solvent properties on the flavonoids quantification was found to be critical. A precipitate was formed once the extracts cooled down. If removed, a significant fraction of the high temperature extracted flavonoids (as much as 71%, at 180 °C) was lost. Such a condition affected especially those compounds that show extremely low solubility in water at room temperature, whereas quercetin glycosylated derivatives were less affected by the polarity change of the medium induced by the temperature change. It was demonstrated that it is necessary to re-dissolve the subcritical water extracts by the addition of ethanol, which led to a medium with a polarity equivalent to that obtained with water at high temperature. At 145 °C, quercetin (15.4 ± 0.4 mg/g DOSW) and quercetin-4′-glucoside (8.4 ± 0.1 mg/g DOSW) accounted for the 90% of the total flavonoids identified. By recovering high added value bioactive compounds from OSW the principles of circular economy were fulfilled, providing a new use for this agricultural waste.

## 1. Introduction

Nowadays, the society is immersed in the transition from a linear to a circular economy, in which the value of products, materials, and resources is maintained in the economy for as long as possible, and the generation of waste is minimized. This means that the byproduct of a process becomes the input of a new one where it acquires new value. More specifically, the European Commission adopted a circular economy action plan *“Closing the loop—An EU action plan for the Circular Economy, COM (2015) 614”* [1], in the year 2015. In this plan, food waste and bio-based products (those based on biological resources) were identified as two of the main priority areas. Among others, onion (*Allium cepa* L.) offers great potential for valorization because it is the second most important horticultural crop worldwide, with a steadily increased production within the last years [2]. For instance, Spain, in the year 2018 produced 1.27 Mt, according to FAO [3]. The onion industry generates every year more than 500,000 t of onion skin waste (OSW) worldwide [4], including skins (the outermost layers), roots, and bulbs unfit for consumption. This has become an environmental concern because onion residues are not suitable for fodder because of their characteristic smell, nor can they be used as an organic fertilizer as is traditionally done, due to the development of phytopathogenic agents [5]. The non-edible brown skin and external layers of onions are rich in phenolic compounds, mainly flavonoids such as quercetin (QC) [2], and quercetin derivatives, which are high-added value natural antioxidants [4]. Beesk et al. [6] pointed out that the distribution and presence of flavonoids in the onion strongly depend on the layer, besides the color and the type of bulbs [7]: The outermost layers are rich quercetin aglycone, whereas the inner part is rich in quercetin glycosides. In total, almost 180 different glycosides of quercetin have been described in nature [8], being the main quercetin glycosides quercetin 4′-O-β-glycoside (QC4′), quercetin 3,4′-O-β-diglycoside (QC3,4′), and quercetin 3-O-β-glycoside (QC3), which represent about 80% of the total flavonoids content. The main drawback of quercetin and quercetin derivatives is the limited solubility in water, which limits their oral bioavailability [9] and extractability. Quercetin glycosides have higher bioavailability because they are better absorbed in the small intestine [10], thanks to the increasing hydrophilicity induced by the glycosylation of at least one of the hydroxyl groups [11,12]. Phenolic compounds can be found in free, esterified, and bound forms. Bound phenolics result to be insoluble since they are covalently bound to cell wall structural components such as cellulose, hemicellulose, lignin, pectin, and proteins [13]. According to Chu et al. [14], onions have approximately 10% of bound phenolics. Therefore, in order to increase its solubility and recovery rate from onion skins, an organic solvent, such as ethanol or methanol, is commonly used. These mixtures of water plus alcoholic solvents in different proportions have been extensively used in conventional extraction processes carried out in stirred tanks [15], or intensified extraction methods such as ultrasound assisted extraction processes [16], or microwave assisted extraction processes [17]. All these extraction methods based on the use of organic solvents require a complex downstream processing in order to remove them. Alternatively, subcritical water can be used to extract flavonoids from onion skins, since subcritical water has been extensively used to extract high added value compounds from natural resources, as summarized by Benito-Roman et al. [18]. Subcritical water refers to water at temperatures ranging from 100 °C (boiling point) to 374 °C (critical point) which remains in a liquid state due to the application of pressure. Changes in the working conditions change the properties of the subcritical water (among them, viscosity, surface tension, and dielectric constant), enhancing mass transfer and the extractability of barely water-soluble bioactive compounds, since subcritical water favors the hydrolysis of the bonds between phenolic compounds and the vegetable matrix.

It is possible to find some works in the literature that deal with the extraction of flavonoids from onion skin wastes using subcritical water [4,19,20,21]. These works studied the extraction process in batch extractors, covering temperatures from 110 to 230 °C and extraction times no longer than 30 min. All of these authors reported a significant increase in the extraction yield compared to the conventional extraction processes. However, one of the main drawbacks of batch extractors is the difficulty to control the heating/cooling times that may result in a too long exposure to the high temperatures, inducing the thermal degradation of the bioactive molecules. As an alternative, a semicontinuous extractor based on the continuous pumping of fresh solvent through the raw material is proposed in this work. Moreover, in those works it is often missed a kinetic modelling of the extraction experiments and a discussion about how the change in the physical properties of water when heated and cooled after the extraction affects the solubility of the target compounds. This change in the solubility may be biasing the identification and quantification process.

The aim of this work was to study the extraction of flavonoids from OSW in an attempt to valorize this extensively agricultural byproduct, using subcritical water in a wide range of temperatures, from 105 to 180 °C, using a semicontinuous extractor equipped with a novel heating system. It was also an aim of this work to study the influence the solvent properties have on the extraction of the flavonoids, and thus, a complete characterization of the extracts in terms of flavonoids identification and quantification is carried out.

## 2. Materials and Methods 

### 2.1. Sample Preparation 

Onion (*Allium cepa* L. cv. Horcal) wastes were collected from the factory of blood sausages *“Embutidos Cardeña”* (Burgos, Spain), which have onion as one of the main ingredients. Onion wastes were processed manually in order to separate the brown skins, which were dried at room temperature. Subsequently, they were milled using a SM100 Retsch mill, equipped with a 1 mm sieve. The raw material was kept at 105 °C for 24 h in order to measure the moisture content (9.45 ± 0.23%), according to the NREL procedure (NREL/TP-510-42621) [22]. All the results obtained in this work were presented per gram of dry OSW (DOSW). The milled onion was stored at 4 °C until the extraction experiments were done.

### 2.2. Analytical Methods

#### 2.2.1. Total Phenolics Content (TPC)

The total phenolic content was measured three times for each sample by the Folin–Ciocalteau method, described by Alonso-Riaño et al. [23], and the average result calculated: A sample of 0.1 mL was mixed with 2.8 mL of water and 0.1 mL of Folin–Ciocalteau’s reagent. After vortexing the solution, 2 mL of sodium carbonate in aqueous solution (7.5% *w/w* sodium carbonate) were subsequently added. After shaking, the mixture was incubated at room temperature in a dark place for 1 h. The absorbance was measured at 750 nm using a V-750 spectrophotometer (Jasco Corporation, Japan). Different concentrations of gallic acid were used to construct the standard curve and final results were expressed as milligrams of gallic acid equivalents per gram of dry OSW used in the extraction *(mg GAE/g DOSW)*.

#### 2.2.2. Total Flavonoids Content (TFC)

The total flavonoids content was determined three times for each sample according to the following procedure, presented by Chang et al. [24], and the average result calculated: 0.5 mL of the sample were mixed with 1.5 mL of absolute ethanol, 0.1 mL of CH_3_COOK solution (0.1 M), 0.1 mL of AlCl_3_ solution (10%, *w/v*), and 2.8 mL of distilled water. Samples were incubated for 30 min and, after being filtered (0.45 μm pore size), the absorbance was measured at 415 nm (spectrophotometer V-750, Jasco Corporation, Japan). A quercetin standard curve in ethanol was used to calculate the TFC of the samples, which was expressed as milligrams of quercetin equivalent per gram of dry OSW *(mg QE/g DOSW)*. Due to the intrinsic color of the samples, a blank standard was also measured. In this case, instead of adding 0.1 mL of the reagent AlCl_3_, 0.1 mL of water were used, the rest of the reagents being equally added. After 30 min of incubation, the absorbance was measured and was subtracted from the sample absorbance readings. 

#### 2.2.3. Antioxidant Activity (AA). FRAP Assay

The total antioxidant activity was measured by the Ferric Reducing Antioxidant Power (FRAP) method developed by Benzie and Strain [25]. The working FRAP solution was prepared by mixing buffer acetate (pH 3.6), 10 mM of 2, 4, 6-tripyridyl-s-triazine (TPTZ) solution and 20 mM FeCl_3_ solutions in the ratio 10:1:1. Then, 2.85 mL of the working FRAP reagent was added to 0.15 mL of the sample and incubated at 37 °C for 30 min. Absorbance was read at 593 nm in a V-750 spectrophotometer (Jasco Corporation, Japan). As standard, a solution of FeSO_4_·7H_2_O was used. Different concentrations of this solution were used for the calibration curve. Results were expressed in *mg FeSO4/g DOSW*.

#### 2.2.4. Characterization of Phenolics and Flavonoids in Extracts

Samples after extraction were characterized by High Performance Liquid Chromatography using a Diode Array Detector (HPLC-DAD, Agilent 1100, CA, USA) with a Kinetex^®^ Biphenyl column (250 × 4.6 mm, particle size 5 µm, pore size 100 Å) supplied by Phenomenex (CA, USA), as described by Alonso-Riaño et al. [23]. The mobile phase consisted of (A) ammonium acetate 5 mM with acetic acid (1%, *v/v*) in water and (B) ammonium acetate 5 mM with acetic acid (1%, *v/v*) in acetonitrile. The gradient profile was the following: From 0 to 7 min, 2% of solvent B (isocratic); from 7 to 20 min, from 2% to 8% solvent B; from 20 to 35 min, from 8% to 10% solvent B and from 35 to 55 min, 10% to 18% solvent B; from 55 to 65 min, increase from 18% to 38% of solvent B; from 65 to 75 min increase up to 65% of solvent B; from 75 to 80 min increase to 80% of solvent B. Post time was 10 min. The flow rate was set at 0.8 mL/min and temperature column was 25 °C. Samples were filtered (0.45 μm pore size) before injection. Three wavelengths were simultaneously used for sample characterization: 280 nm, 330 nm, and 370 nm. ChemStation software was employed to collect and analyze the chromatographic data delivered by the diode array detector and our own library was used to identify the different polyphenols by comparing retention times and UV spectrum with those of standards, as shown in Table S1 of supplementary material, together with example of a chromatogram obtained (Figure S1).

The total quercetin equivalent (Total QCE) was calculated as the sum of QC, QC4′, QC3, and QC3,4′ expressed in *mg quercetin equivalents (QCE)/g DOSW*, according to Equation (1), provided by Jang et al. [16]:

(1) Total QCE (mgg DOSW) = QC + QC4′·MWQCMWQC4′ + QC3·MWQCMWQC3 + QC3,4′·MWQCMWQC3,4′

where QC, QC4′, QC3 and QC3,4′ are the contents in OSW expressed in mg/g DOSW and M_w_ refers to the molecular weight of the respective flavonoids.

### 2.3. Conventional Extraction

The conventional extraction experiment was carried out in the conditions found as optimal in a previous work: 37 °C for 60 min using as solvent ethanol:water mixture (70%, *v/v*). 10 g of OSW were transferred to an Erlenmeyer flask, where 100 mL of solvent were added. The flask was placed in an incubator shaker (Model G25, New Brunswick Scientific Co., NJ, USA) and stirring was set at 275 rpm. After the extraction, the solid liquid mixture was separated under vacuum filtration. Solids were discarded and the liquid extract was kept at 4 °C until analysis.

### 2.4. Subcritical Water Extraction

**Figure 1:**
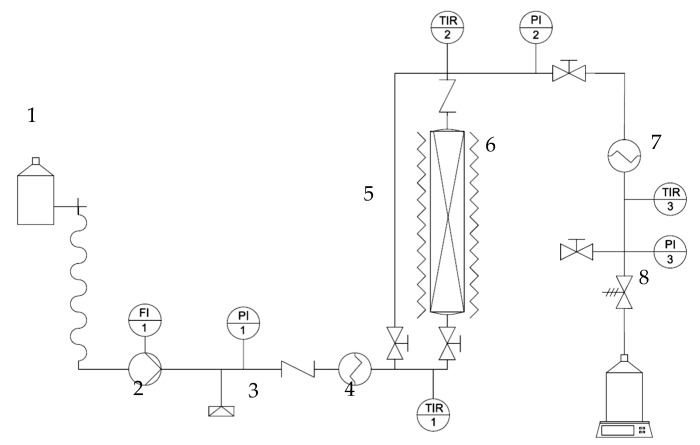
Subcritical water laboratory scale extraction plant used in this work. (1) Solvent bottle; (2) pump; (3) bursting disk; (4) heater; (5) by-pass pipe; (6) extractor; (7) chiller; and (8) backpressure regulator.

The extraction experiments using subcritical water were carried out in a semi-continuous laboratory scale plant as shown in Figure 1. Essentially, this plant consists of a 27 mL extractor (Autoclave Engineers, Erie, PA, USA), an HPLC pump (Gilson 305, SC-10 head) to pump the solvent through the extractor and a back-pressure regulator, to keep the working pressure at 5 MPa. The plant is equipped with pressure gauges and temperature indicators in three different places: At the inlet and outlet of the extractor and right before the backpressure regulator. All three are connected to the software acquisition data PicoLog. The heating system of this plant is double: The solvent is heated right after the pump by means of a band heater to get the desired temperature at the inlet of the extractor, and another band heater around the extractor keeps such temperature. A 20 cm long (diameter 5 cm) band heater is used in order to keep the temperature in the extractor.

In a typical experiment, around 4 g of OSW were placed in the extractor mixed with around 8 g of 5 mm glass beads in order to avoid packaging and channeling through the bed. Subsequently, the heating band was put around it; finally, the extractor was placed in the extraction plant. Then, the whole system was filled up with water and pressurized. After that, water was heated by means of the first band heater: It was continuously pumped through it at the working flow rate, but bypassing the extractor. Once water reached the working temperature, bypass valve was closed, so water was continuously pumped through the extractor. The band heater around the extractor helped to keep the desired working temperature along the experiment. The extract was cooled down in a chilled water bath right after exiting the extractor and kept at 4 °C until analysis. All the extraction experiments lasted 180 min, and the subcritical water experiments were done in a temperature range from 105 to 180 °C. Some initial experiments were done in order to select the suitable flow rate. This working procedure, in which only water at the extraction temperature is passed through the extractor, minimizes the time the raw material is exposed to the high temperature, preserving the integrity of the bioactive compounds.

The total extraction time was split in nine intervals (0–10 min; 10–20 min; 20–30 min; 30–45 min; 45–60 min; 60–80 min; 80–110 min; 110–140 min; and 140–180 min). The extracts collected in each interval were analyzed and then, the accumulated extraction curve calculated.

#### Subcritical Water Extraction Modelling

The extraction kinetic curves obtained for the three different responses studied (TPC, TFC, and AA-FRAP) were fitted to two empirical models used by Alonso-Riaño et al. [23] to model the extraction of phenolic compounds. The first empirical model proposed was the Power Law Model, which is presented in Equation (2). *B* is a constant incorporating the characteristics of the particle-active substance system and *n* is the diffusional exponent, with values lower than 1 for most vegetable materials. One of the main features of this model is that it does not approach to a limit with time.

(2) $${\mathrm{Response}=B\cdot t}^{n}$$

The second empirical model, presented in Equation (3) is the Weibull model; where *t* is the extraction time, *A* is a kinetic parameter that represents the maximum extraction yield at infinite extraction time and *k* is a kind of extraction rate constant; *n* is the shape parameter of the extraction curve.

(3) $$\mathrm{Response}=A\cdot \left[1-\exp\left(-{k\cdot t}^{n}\right)\right]$$

To estimate the kinetic parameters, non-linear regression was performed by using the Marquardt algorithm (Statgraphics 18-X64). Experimental results were then compared to those of the model prediction through the values of the Root Mean Square Deviation (RMSD), calculated according to Equation (4):

(4) $$\mathrm{RMSD}=\sqrt{\frac{\sum_{i=1}^{n}{\left({\mathrm{Response}}_{\exp}-{\mathrm{Response}}_{\mathrm{calc}}\right)}^{2}}{n}}$$

### 2.5. Statistical Analysis

The statistical analysis of the results was done by means of Statgraphics 18-X64, considering 95% confidence level.

## 3. Results

### 3.1. Extraction Experiments

#### 3.1.1. Conventional Extraction: Ethanol/Water 70% (*v/v*)

Results of the conventional extraction experiment are shown in Table 1. These results were used as reference in order to assess the suitability of subcritical water to extract bioactive compounds from OSW.

#### 3.1.2. Subcritical Water Extraction

##### Effect of the Flow Rate

**Figure 2:**
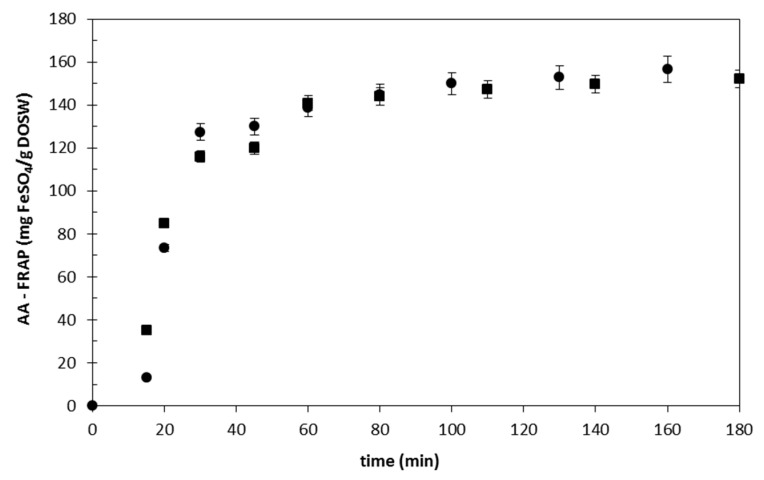
Accumulated antioxidant activity of the extracts obtained from onion skin wastes (OSW) (150 °C and 5 MPa) as a function of the working flow rate (●, 2.5 mL/min; ■, 6 mL/min).

Figure 2 presents the accumulated antioxidant activity of the extracts obtained with subcritical water at 150 °C, 5 MPa and two different flow rates (2.5 and 6 mL/min). It can be clearly seen that, after 80 min, the antioxidant activity of the extracts is not increasing, what indicates that the extraction of bioactive compounds is almost concluded. In general, three different periods can be observed in a fixed bed extraction curve: (1) The cell structure swells (extract not readily accessible for the solvent), (2) the maximum extraction rate (3) solid depletion, no more extraction of bioactive compounds is achieved, despite the increase in the extraction time. These three periods can be identified in Figure 2, corresponding the first 10 min of the extraction to the first period, then the maximum extraction rate until minute 30, and finally decreasing the extraction rate to reach a plateau at about 80 min of extraction. The extraction done at 6 mL/min showed faster extraction rate within the first 15 min, but after that period, no differences in the extraction rate were observed for the two flow rates studied. Similar trends were observed for the analysis of TPC and TFC.

The maximum extraction yield obtained at 2.5 mL/min was 94.7 ± 2.7 mg GAE/g DOSW and 25.3 ± 1.1 mg QE/g DOSW and AA was 156.7 ± 6.2 mg FeSO4/g DOSW. When the flow rate used was 6 mL/min, the following results were obtained: 80.3 ± 3.9 mg GAE/g DOSW, 25.7 ± 0.6 mg QE/g DOSW and AA was 152.2 ± 4.1 mg FeSO4/g DOSW. No statistically significant effect of the flow rate was detected. Therefore, the lowest flow rate was selected, in order to reduce the amount of solvent consumed and avoid the dilution of the extracts. The increase of the flow rate did not improve the extraction rate, indicating that, under these conditions, external mass transfer does not limit the extraction [26].

##### Effect of the Extraction Temperature

**Figure 3:**
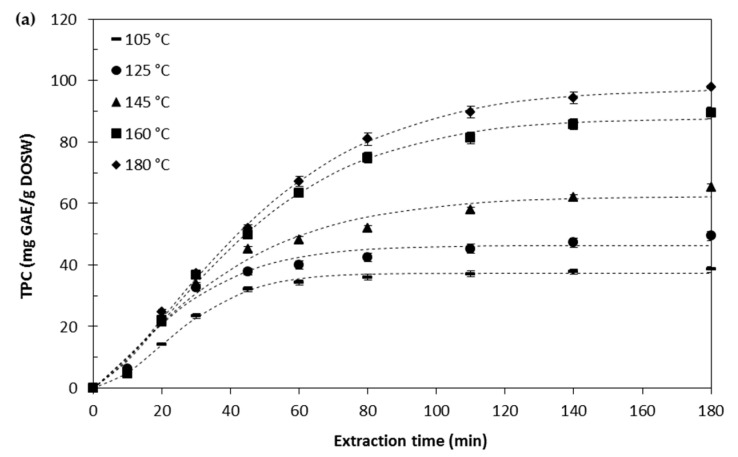

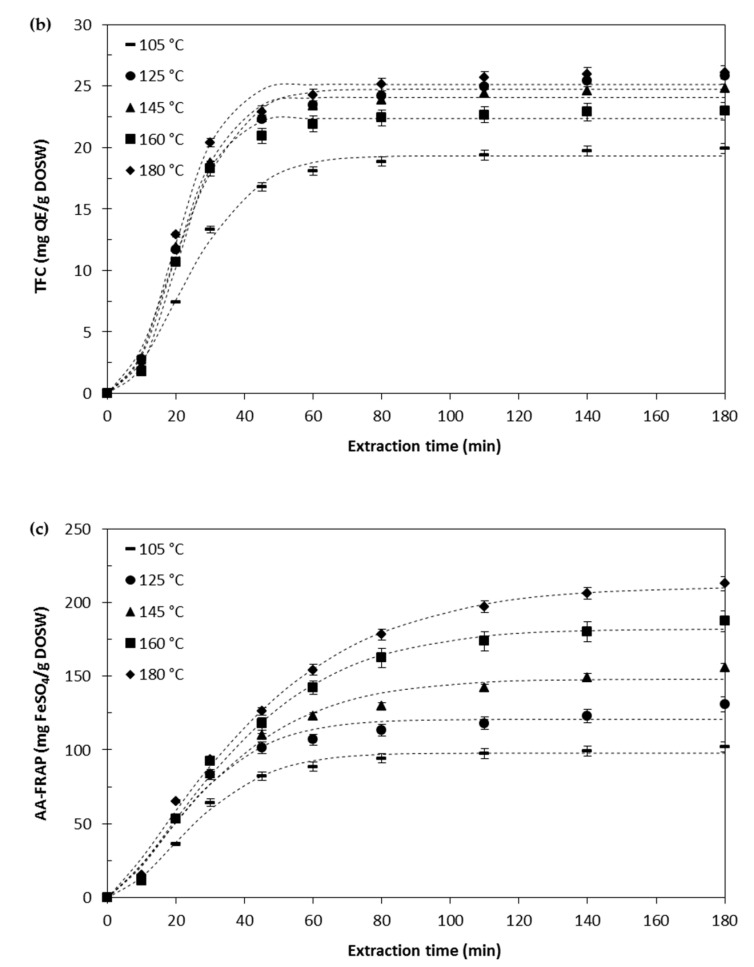
Accumulated extraction curves at different temperatures for TPC (**a**), TFC (**b**), and antioxidant activity (**c**) obtained from onion skin wastes at 2.5 mL/min and 5 MPa. Dashed lines represent the extraction curve obtained with the Weibull model.

The extraction temperature in the range 105–180 °C, using a flow rate of 2.5 mL/min was studied. A color change of the extracts with temperature was observed, getting darker the higher the temperature and the longer the extraction time. This color change may be due to the formation of sugar degradation products as a consequence of the Maillard reactions. Regarding the TPC of the extracts, the higher the temperature the higher the TPC in the extract: At 180 °C the maximum TPC is 97.8 ± 2.1 mg GAE/g DOSW, whereas at 105 °C this value is only 38.5 ± 0.9 mg GAE/g DOSW. At the lowest temperatures (105 and 125 °C), the extraction of TPC remains almost constant after 60 min of extraction, whereas at higher temperatures, the extraction of phenolic compounds tends to increase for longer times, as can be seen in Figure 3a.

The extraction of flavonoids (Figure 3b), allowed to draw two main conclusions: First, it is a very fast extraction, after 45 min almost 95% of the total flavonoids extracted after 180 min had been extracted, being the period from 10 to 30 min the one that provided the highest flavonoid recoveries. Second, apparently the effect of temperature is limited. Only at 105 °C it was possible to detect a significantly lower extraction yield, 19.9 ± 0.4 mg QE/g DOSW, compared to the results obtained at higher temperatures, that provided extraction yields around 25 mg QE/g DOSW.

The antioxidant activity (Figure 3c) keeps increasing with the extraction temperature and the extraction time, until 110 min, when a plateau was reached. In this case, it is possible to see that the temperature is favoring the extraction of bioactive compounds with antioxidant activity. The part of the extraction curve in which the higher extraction yield is happening is kept for longer times the higher the temperature.

The results presented in Figure 3a–c, allow to conclude that, after 45 min of extraction, subcritical water provides extracts with higher antioxidant activity than those obtained in the conventional extraction process at 37 °C using aqueous ethanol (70%, *v/v*) as solvent, regardless the temperature. The extraction of bioactive compounds is affected by the properties of the solvent. Water and ethanol are perfectly miscible solvents but with different polarity. At 37 °C, the dielectric constant moves from 74.3 (value that corresponds to pure water) to 22.5, value that corresponds to pure ethanol. The dielectric constant of the 70% (*v/v*) ethanol/water mixture was calculated according to the equation provided by Fakhree et al. [27] and resulted to be 37.6. When subcritical water was used, the dielectric constant moved from 53.9 (105 °C), 49.1 (125 °C), 44.7 (145 °C), 41.6 (160 °C), to 37.9 (180 °C). It is possible to see that ethanol/water mixture (70%, *v/v*) has a dielectric constant similar to water at 180 °C; however, the extraction of TPC was higher when subcritical water at 180 °C was used, what indicated that subcritical water enhances and promotes the extraction of bioactive compounds. Moreover, increases in temperature induce increases in the ionic product of water (K_w_), which implies that the pH changes from 7.0 at room temperature to about 5.7 at 180 °C, resulting in higher ionic strength of hydronium and hydroxide ions than at ambient temperature [28]. Therefore, the higher ionic strength and lower pH may induce the acid hydrolysis of the bonds between the bioactive compounds and the solid matrix, promoting their release. It is also expected that the increase in the absorbance for the samples obtained using subcritical water may also be influenced by the presence of compounds formed because of the Maillard reactions, which may interfere with the Folin–Ciocalteau reagent.

In general, the comparison of the results obtained in this work with others that can be found in the literature is hard, due to differences in the experimental procedures, reactor configurations and onion cultivars. Lee et al. [19] studied the recovery of quercetin and derivatives from onion peels, by using subcritical water at different temperatures (110–165 °C) for 15 min in a batch extractor and conventional extraction with ethanol (70%, *v/v*) at 60 °C for 3 h. They found that the extracts obtained with subcritical water had up to four times higher antioxidant activity than extracts obtained using ethanol. Water at 110 °C, also provided extracts with higher TPC (38.8 ± 1.0 mg GAE/g onion peel) and TFC (21.2 ± 1.9 mg QE/g onion peel) yields than those obtained with ethanol (TPC = 16.6 ± 0.3 mg GAE/g onion, TFC = 2.8 ± 0.1 mg QE/g onion peel). When temperature was 165 °C, TPC (11.3 ± 0.5 mg GAE/g onion peel) and TFC (5.4 ± 0.4 mg QE/g onion peel) values were not so different from those obtained with ethanol. The best results were obtained at 110 °C, conditions that led to a higher productivity per time unit, since subcritical water extraction only lasted 15 min whereas conventional extraction experiments took 3 h. The increase in the AA might be biased because of the presence of compounds such as 5-hydroxymethylfurfural (5-HMF), formed at high temperature due to the Maillard reactions. These compounds are known to exhibit certain antioxidant activity [29]. However, Munir et al. [4] did report a significant increase in the extraction of flavonoids and phenolics compared to the conventional extraction processes. These authors studied the extraction of flavonoids from onion skin in a batch extractor using subcritical water (170–230 °C, 3 MPa, 30 min). They found a maximum TFC of 114.8 mg QE/g onion obtained at 170 °C, 30 min and 3 MPa (pH was set at 10, and particle size 100–200 μm), in a batch reactor in which 12 g of sample were loaded and 600 mL of solvent were used. This value resulted to be significantly higher than the results presented in our work, without a clear reason for that difference. Ko et al. [20] worked at temperatures in the range 100–190 °C, at pressures in the range from 9 to 13 MPa in processes that lasted up to 30 min. These authors found that temperatures and times higher than 165 °C and 15 min did not increase the recovery of flavonoids. Compared to the conventional extraction experiments based on the use of organic solvents, these authors demonstrated that subcritical water increased the extraction yield of quercetin up to 8 times the amount obtained when ethanol or methanol at 60 °C was used (the extraction lasted up to 2 h). The increase in the quercetin extraction yield was about four times higher when subcritical water at 165 °C was used (16.29 ± 0.75 mg quercetin/g onion skin) compared to water at 100 °C for 3 h. Kim et al. [21] presented a combined extraction system composed by subcritical water extraction (145 °C and 15 min) coupled with intense pulse light (1200 V for 60 s). This extraction system allowed to recover quercetin, 17.32 ± 1.12 mg quercetin/g onion skin, which was slightly higher than the amount recovered when only subcritical water (145 °C, 15 min) was used (15.19 ± 1.12 mg quercetin/g onion skin), in this batch pressurized tank (ratio 25 g of onion skins in 1.1 L of water). The range of temperature studied in this work was 105 to 185 °C.

##### Kinetic Modelling

Table 2 summarizes the parameters obtained for the two models tried in this work. It is possible to seen that the Weibull model provided better results than the power law model, since the RMSD values were lower. The experimental data for all the three responses fitted perfectly to the Weibull model whereas the power law model did not work well. This fact has to do with the nature of the power law model, which does not approach to a limit with time, and according to Figure 3a–c, the studied responses tend to reach a constant value with time.

### 3.2. Polyphenols Identification

#### 3.2.1. Conventional Extraction: Ethanol/Water 70% (*v/v*) Experiment

QC4′ and QC were the main phenolic compounds extracted (9.8 ± 0.3 mg/g DOSW and 6.6 ± 0.2 mg/g DOSW, respectively). Other quercetin glucosides, such as QC3,4′ and QC3 had a concentration of 2.04 ± 0.03 mg/g DOSW and 0.21 ± 0.03 mg/g DOSW, respectively. In total, the sum of quercetin and its derivatives was 18.6 ± 0.5 mg/g DOSW, which resulted to be 14.1 ± 0.4 mg/g DOSW in terms of total quercetin equivalents, calculated according to Equation (1). Under these experimental conditions, neither kaempferol nor isorhamnetin were detected.

#### 3.2.2. Subcritical Water Experiments

As described in Section Effect of the Extraction Temperature, the extraction of flavonoids is very fast, happening exclusively within the first 60 min of extraction. In this work it has been possible to identify and quantify the following phenolic compounds: Protocatechuic acid, p-hydroxybenzoic acid, vanillinic acid, p-cumaric acid, QC, QC4′, QC3,4′, QC3, myricetin, kaempferol, isorhamnetin, QC-3-rhamnoside and isorhamnetin-3-glucoside.

##### The Effect of the Medium on the Phenolic Compounds Solubilization

**Figure 4:**
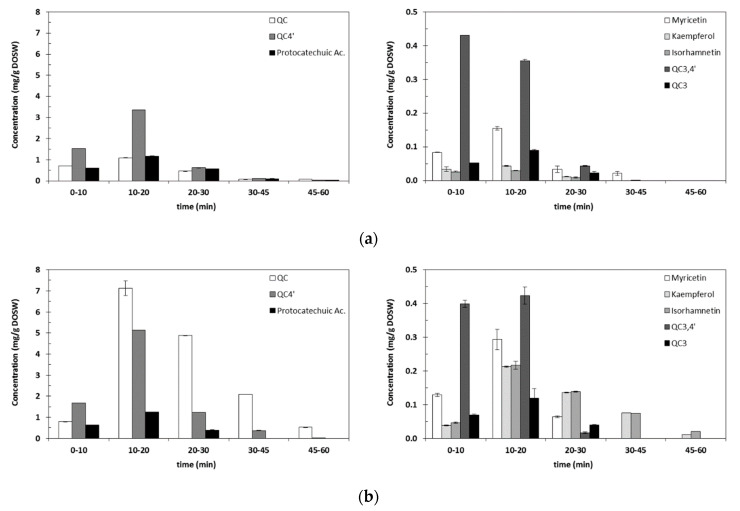
Flavonoids quantification after the extraction at 145 °C; (**a**) soluble fraction obtained at 145 °C as obtained after centrifugation. (**b**) After the addition of ethanol to the aqueous extracts.

One of the main features of subcritical water is the ability to modify its properties when changing pressure and especially temperature: Density, surface tension, viscosity, and diffusion change dramatically enhancing mass transfer. A temperature increase also contributes to weaken the hydrogen bonds between the bioactive compounds and the solid matrix, accelerating the compound desorption. The dielectric constant of water also changes in a great extent when heated: At room temperature (25 °C), it has a value of 78.6, but decreases dramatically, down to 53.9 at 105 °C or 37.9 at 180 °C. This change in the polarity involves changes in the compounds solubility, leading to the irreversible precipitation at room temperature of compounds that were dissolved during the extraction at high temperatures. If prior to analysis, samples are centrifuged and filtered in order to remove that precipitate, part of the compounds extracted under the high temperature conditions are removed; thus, quantification of compounds will be biased and will not describe the real outcome of the high temperature extraction. For instance, quercetin has a solubility in water of 2.15 ppm at 25 °C, which is increased up to 665 ppm at 140 °C [30]. Therefore, in order to overcome this limitation and prove the importance the decrease in solubility has on the identification and quantification of bioactive compounds extracted at high temperatures, the extracts obtained at 145 °C and 5 MPa were analyzed in two different ways: As obtained (centrifuged and filtered to remove the precipitate formed after cooling down, so the soluble fraction can be quantified) and ethanol added (final concentration of the extract, 60% ethanol, *v/v*) in order to completely dissolve the precipitate formed and quantify the total phenolics extracted. An ethanol/water mixture (60%, *v/v*) has a dielectric constant around 43, which is a value close to dielectric constant of water at 145 °C (44.7). Results are shown in Figure 4.

Figure 4a,b, show that the extraction of flavonoids mainly occurs within the first 30 min. The increase in the amount of flavonoids detected with the addition of ethanol to solubilize the precipitate formed can also be observed. The observed increase was higher the lower the solubility of these compounds in water. From lowest to highest, the solubility expressed in g/L is: Isorhamnetin (0.15) < kaempferol (0.18) < QC (0.26) < myricetin (0.3) < QC4′ (1.59) < QC3 (1.95) < QC3,4′ (4.59) < ac. Protocatechuic (12.4) [31]. The highest increase in the amount detected was found for isorhamnetin (7.7 times higher), followed by QC (6.4 times), kaempferol (5.3 times), myricetin (1.66 times), QC4′ (1.5 times), QC3 (1.4 times), QC3,4′ (the same amount), and ac. protocatechuic, the solubility of which decreases with the addition of ethanol, therefore decreasing the amount detected (0.9 times lower).

Once it was proved that in order to get accurate results of the compounds extracted using subcritical water, it is important to find a way to re-dissolve them, the extracts obtained at the other temperatures were re-dissolved in ethanol and analyzed. In Table 3, results regarding the soluble fraction and the total compounds extracted (determined after the re-dissolution of the extract after the addition of ethanol), are presented. It is possible to see that, if only the soluble fraction is quantified, the total amount of extracted flavonoids is underestimated. At 105 °C the soluble fraction represents 40.6% of the total quercetins extracted, whereas at 145 °C is 36.5% and at 180 °C it is only 28.9%, which indicates that high temperature promotes the extraction of water insoluble quercetin.

##### Effect of the Temperature on the Bioactive Compounds Extracted

**Figure 5:**
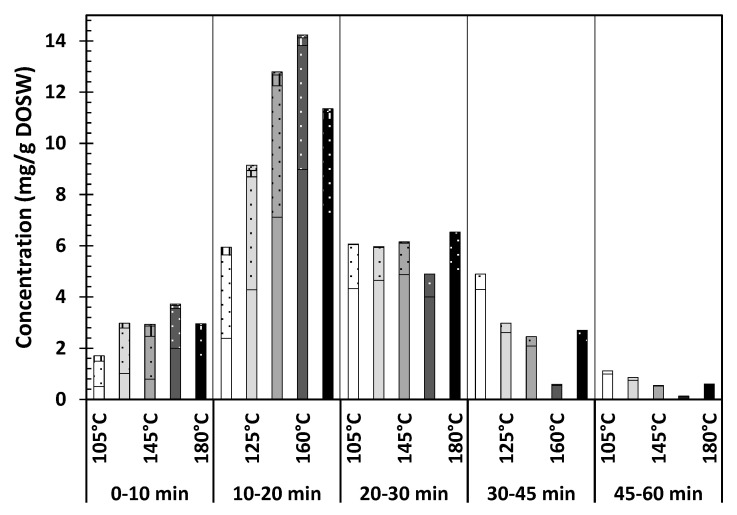
Total quercetins extracted as a function of the extraction time and the temperature. Solid color refers to QC; dotted to QC4′; vertical lines to QC3,4′ and diagonal lines to QC3.

According to the results shown in Table 3, it is possible to see that temperature affects the extraction of flavonoids and the best results in terms of total quercetins extracted (the sum of QC+QC4′+QC3,4′+QC3), were obtained at 145 °C: 24.9 ± 0.6 mg/g DOSW, which accounts for the 92% of the total flavonoids detected. Figure 5 presents the total amount of quercetins extracted within the first hour of extraction at each of the studied temperatures. It can be seen that the extraction is fast, happening mainly in the period from 10 to 20 min of extraction time, decreasing the extraction rate from that moment. It is at 160 °C that the highest recovery happens in this period, but in total at 145 °C slightly more quercetins are recovered after 60 min of extraction. In all the conditions QC is the main compound extracted followed by QC4′. The extraction of QC3,4′ happens mainly within the first 20 min, whereas QC3 is barely extracted.

Ko et al. [20] reported 16.3 ± 0.8 mg QC/g onion (8 times higher than the obtained with ethanol 60 °C) and 3.15 mg QC4′/g onion for extractions done at 165 °C and 15 min. In total, the sum QC+QC4′ accounted for the 99% of the total flavonoids present in the extract and approximately 92.4% of the total QC was recovered from the original material.

More specifically, according to Table 3, at 145 °C, QC aglycone concentration was 15.4 ± 0.4 mg/g, which represents 62% of the total quercetins identified, whereas QC4′ concentration was 8.4 ± 0.12 mg/g onion. Further increases of temperature involved higher share of quercetin aglycone of the total quercetins detected: At 180 °C QC aglycone represented 68% of the total. These results are in agreement with other authors who reported that, in the onion skin, QC glucosides are barely found [32], since 67–86% of the total QC is in the aglycone form [21]. QC is the main flavonoid in the external layers of the onion, because it derives from the deglycosilation of the QC glucosides as a consequence on the intrinsic skin formation process [33]. It has the very specific role of protecting the onion from the UV radiation, given the fact that quercetin aglycone has higher antioxidant activity than the glycoside derivatives [34]. If the whole onion is used as a source of flavonoids, then QC4′ and QC3,4′ account for the 80% of the total flavonoids extracted [11,15]. The remaining 20% are 17 different components, among them, quercetin-3-o-glucoside and isorhamnetin-3-glucose are the most abundant [35].

QC and QC4′ have been identified as the two major flavonoids extracted from OSW. As can be inferred from the results presented in Table 3, the ratio QC4′/QC did not remain constant throughout the extraction process, moving from the maximum of 0.60 at 125 °C to the minimum detected at 180 °C (0.42). Considering that the total amount of QC increases with temperature and the amount of QC4′ decreases from 145 °C, as presented in Table 3, it seems likely that the high temperatures are favoring the hydrolysis of the quercetin glucoside to yield quercetin aglycone plus glucose. When the total quercetin equivalent values are analyzed, it is possible to see that it increases up to 160 °C, remaining constant, with no statistically significant differences when the temperatures are further increased. Ko et al. [36] also indicated that the aglycone form of the flavonoids has increasing stability with increasing temperature in subcritical water extraction experiments. Wianowska and Dawidowicz [37] reported the effect of the solvent on the conversion of QC4′ to QC. These authors indicated that when the extraction of flavonoids is done using water/methanol mixtures, an increase in the percentage of water in the mixture induces the hydrolysis of QC4′ to QC aglycone.

According to Slimestad et al. [38] 19–21% of the total flavonoids in onion are kaempferol, isorhamnetin and myricetin derivatives, flavonoids that are highly insoluble in water [39], which is in agreement with the results presented in Table 3. The presence of kaempferol is temperature sensitive, with a maximum obtained at 145 °C (0.46 ± 0.01 mg/g DOSW). This temperature sensitivity was in agreement with the results published by Munir et al. [4], who detected kaempferol at 170 °C but not at 230 °C. Similar results were obtained by Piechowiak et al. [40], who extracted flavonoids from onion skins using methanol as solvent. The extraction of these compounds was affected by temperature (increasing up to a maximum detected at 44 °C), processing time and the nature of the solvent used. These authors identified the following flavonoids: QC (315.6 mg/g extract), QC3 (40.3 mg/g extract), isorhamnetin (14.8 mg/g extract), and kaempferol (10.9 mg/g extract). Sagar et al. [41] reported kaempferol in concentrations of 0.124 to 0.710 mg/g onion, strongly affected by the onion variety, whereas Miean and Mohamed [42] reported 0.832 mg kaempferol/g onion leaves).

Similar trend was observed for isorhamnetin, flavonoid that was extracted in similar quantities as kaempferol. The extraction of kaempferol and isorhamnetin also happened mainly within the first 20 min of extraction, decreasing from that moment and being almost negligible from minute 45 to 60. Ko et al. [36] also reported similar behavior for these two flavonoids at 190 °C, with an increase in the amount extracted up to 15 min of extraction. However, according to Table 3 myricetin exhibited different trend: The amount extracted increased up to 125 °C, then decreased with a minimum at 160 °C (0.47 ± 0.02 mg/g DOSW), subsequently increasing to a maximum at 180 °C (0.601 ± 0.016 mg/g DOSW). Søltoft et al. [43] also identified isorhamnetin derivatives and myricetin as flavonoids present in onions. In our case, at 145 °C, isorhamentin-3-glucoside was only detected in the soluble fraction with a concentration of 0.061 ± 0.004 mg/g DOSW (145 °C, decreasing with temperature). Slimestad et al. [38] indicated that 80–85% of the flavonoids are QC derivatives and the 19–21% are kaempferol, isorhamnetin and myricetin derivatives. These authors studied the influence of the onion variety on the nature of the compounds extracted: Red onion contain more QC (334 vs. 214 mg/kg onion), kaempferol (11 vs. 6 mg/kg onion), and myricetin (27 vs. 0.2 mg/kg onion) than yellow onion; but on the other hand, isorhamnetin was more abundant in yellow onion than in red onion (50 vs. 43 mg/kg onion). Prakash et al. (Prakash, Singh, and Upadhyay, 2007) using methanol at solvent and performing the extraction overnight at room temperature, reported quercetin and kaempferol concentrations of 5.1 mg/g onion and 0.48 mg/g onion respectively. Ko et al. (Ko et al., 2011) reported the presence of kaempferol (0.88 ± 0.23 mg/g onion), isorhamnetin (0.74 ± 0.18 mg/g onion) and rutin (0.93 ± 0.15 mg/g onion). In our work, rutin was not found in any of the experimental conditions studied as compared with the standard.

Another quercetin derivative (quercetin 3-rhamnoside, also known as quecitrin) was also detected in the samples extracted using subcritical water as solvent. This compound resulted to be very temperature sensitive, decreasing with temperature (maximum at 125 °C, around 0.1 mg/g OSW), in agreement with the results presented by Ko et al. [36], who reported around 0.013 mg/g dried materials) at 110 °C.

Another of the major phenolic compound detected has been protocatechuic acid (3,4-dihydroxybenzoic acid), which is found in the most external layers of the onion [32], is formed as a consequence of the degradation of quercetin in an autoxidation process [37,44], and is known for having very powerful antioxidant activity [45]. In our work this compound has been identified in all the temperature range essayed, detecting the highest concentration at 160 °C (2.49 ± 0.15 mg/g onion). Further increases of temperature produced a decrease in the amount of this compound that probably suffered degradation to other compounds.

Other compounds found in the extracts and related to protocatechuic acid, are vanillinic, p-hydroxybenzoic and p-cumaric acids (hydroxycinamic acid). These compounds, which concentration is presented in Table 3, can be considered as minor components.

## 4. Conclusions

This is a complete study in which, the major phenolic compounds extracted from onion skin wastes using subcritical water were identified. In a semicontinuous extractor it is possible to time-fractionate the extracts, which allowed to demonstrate that the extraction of flavonoids was relatively fast, happening within the first 60 min of extraction, and more specifically within the very first half an hour. In this study, it was also demonstrated that after the subcritical water extraction, once the extracts are cooled down to room temperature, a precipitate is formed, which, if removed, a significant part of the bioactive compounds dissolved at the high temperature is not considered. Therefore, it is a key step to re-dissolve this precipitate in order to accurately identify and quantify the bioactive compounds extracted under the high temperature conditions. The addition of ethanol to the cooled subcritical water extract, leads to a solvent that has similar polarity to that of water at high temperature. All in all, the extraction of flavonoids resulted to be temperature sensitive, being the most suitable temperature 145 °C, since at higher temperatures, despite the increase in the antioxidant activity of the extracts, the TFC began to decrease. The extraction of high added valuable compounds from this extensively generated by-product, contributes to its valorization, following the principles of the transition to a circular economy: the waste of a process becomes the input of a new one, where it acquires new value.

## Figures and Tables

**Table 1:** Experimental results for the conventional extraction experiments.

TPC (mg GAE/g DOSW)	TFC (mg QE/g DOSW)	AA-FRAP (mg FeSO_4_/g DOSW)
46.7 ± 1.4	20.4 ± 0.2	67.9 ± 1.4

**Table 2:** Kinetic model parameters obtained for the three responses studied at different extraction temperatures.

		**Weibull Model**				**Power Law Model**		
		**A**	**k**	**n**	**RMSD**	**B**	**n**	**RMSD**
**TPC** **(mg GAE/g DOSW)**	**105 °C**	37.3	0.0030	1.70	0.8	5.68	0.39	5.0
	**125 °C**	46.3	0.0122	1.30	2.2	7.85	0.37	5.0
	**145 °C**	62.3	0.0112	1.21	2.9	6.36	0.46	6.0
	**160 °C**	87.8	0.0046	1.38	1.7	5.80	0.55	8.0
	**180 °C**	97.3	0.0045	1.36	1.6	5.81	0.57	8.3
**TFC** **(mg QE/g DOSW)**	**105 °C**	19.3	0.0024	1.79	0.5	3.14	0.38	2.8
	**125 °C**	24.7	0.0022	1.87	0.7	4.76	0.35	3.5
	**145 °C**	24.1	0.0009	2.18	0.6	4.95	0.34	3.8
	**160 °C**	22.4	0.0004	2.49	0.5	4.65	0.34	3.7
	**180 °C**	25.1	0.0008	2.23	0.8	5.32	0.33	3.9
**AA-FRAP** **(mg FeSO_4_/g DOSW)**	**105 °C**	97.8	0.0036	1.64	2.9	14.69	0.40	13.0
	**125 °C**	120.7	0.0062	1.50	5.5	19.28	0.39	14.3
	**145 °C**	148.0	0.0074	1.35	6.0	16.66	0.45	15.7
	**160 °C**	182.1	0.0056	1.38	5.0	16.27	0.49	18.5
	**180 °C**	211.0	0.0074	1.27	4.6	16.53	0.51	18.2

Values higher than 1 for the *n* parameter in the Weibull model indicated that the curve is sigmoidal with upward curvature. Regarding the *k* parameter for the Weibull model, it can be considered as the higher the value, the higher the extraction rate. Values for the *k* parameters obtained for the TFC were much higher than those obtained for the other two parameters, which indicates that the extraction of flavonoids was higher than the extraction of phenolic compounds.

**Table 3:** Composition of the extracts obtained at different temperatures using subcritical water. It is presented the soluble fraction and the total content in phenolic compounds (re-dissolved in ethanol) of the extracts.

	Soluble Fraction (mg/g DOSW)					Total (mg/g DOSW)				
	105 °C	125 °C	145 °C	160 °C	180 °C	105 °C	125 °C	145 °C	160 °C	180 °C
**QC**	2.3 ± 0.1	2.4 ± 0.4	2.4 ± 0.1	2.2 ± 0.1	1.9 ± 0.3	12.5 ± 0.2	13.3 ± 0.1	15.4 ± 0.4	16.0 ± 0.2	16.4 ± 0.6
**QC4’**	5.0 ± 0.2	5.7 ± 0.3	5.7 ± 0.2	4.6 ± 0.1	4.5 ± 0.1	6.7 ± 0.1	8.0 ± 0.2	8.4 ± 0.1	7.8 ± 0.1	7.0 ± 0.1
**QC3**	0.13 ± 0.02	0.15 ± 0.03	0.16 ± 0.01	0.11 ± 0.03	0.14 ± 0.04	ND	0.23 ± 0.01	0.29 ± 0.03	0.245 ± 0.012	0.27 ± 0.01
**QC3,4’**	0.55 ± 0.04	0.50 ± 0.01	0.83 ± 0.09	0.38 ± 0.01	0.47 ± 0.05	0.54 ± 0.04	0.44 ± 0.02	0.84 ± 0.04	0.453 ± 0.015	0.45 ± 0.02
**Kaempferol**	0.11 ± 0.01	0.062 ± 0.012	0.09 ± 0.01	0.06 ± 0.01	0.07 ± 0.01	0.29 ± 0.03	0.34 ± 0.01	0.46 ± 0.01	0.382 ± 0.015	0.31 ± 0.02
**Isorhamnetin**	0.08 ± 0.02	0.08 ± 0.03	0.07 ± 0.01	0.062 ± 0.011	0.07 ± 0.02	0.27 ± 0.02	0.38 ± 0.01	0.48 ± 0.02	0.42 ± 0.02	0.37 ± 0.03
**Myricetin**	0.31 ± 0.05	0.39 ± 0.09	0.29 ± 0.02	0.25 ± 0.05	0.26 ± 0.04	0.52 ± 0.08	0.56 ± 0.09	0.49 ± 0.04	0.47 ± 0.02	0.601 ± 0.016
**Protocatechuic Ac.**	2.0 ± 0.1	2.2 ± 0.1	2.5 ± 0.0	2.4 ± 0.1	2.4 ± 0.1	1.7 ± 0.2	2.0 ± 0.1	2.3 ± 0.3	2.0 ± 0.15	2.29 ± 0.11
**p-Hydroxybenzoic Ac.**	0.16 ± 0.01	0.12 ± 0.05	0.16 ± 0.01	0.07 ± 0.01	0.07 ± 0.02	ND	ND	0.14 ± 0.00	ND	ND
**Cumaric Ac.**	0.16 ± 0.05	0.09 ± 0.01	0.13 ± 0.01	0.06 ± 0.01	0.03 ± 0.01	ND	ND	0.05 ± 0.01	ND	ND
**Vanillinic Ac.**	0.04 ± 0.01	0.04 ± 0.01	0.05 ± 0.01	0.03 ± 0.01	0.03 ± 0.01	ND	ND	ND	ND	ND
Total QCs *	8.0 ± 0.4 ^A,B,C^	8.7 ± 0.7 ^C,D^	9.1 ± 0.4 ^D^	7.3 ± 0.2 ^A,B^	7.0 ± 0.5 ^A^	19.7 ± 0.3 ^A^	22.0 ± 0.3 ^B^	24.9 ± 0.6 ^C^	24.5 ± 0.3 ^C^	24.1 ± 0.7 ^C^
Total QCE **	5.9 ± 0.3 ^A,B,C^	6.4 ± 0.6 ^C,D^	6.6 ± 0.3 ^C^	5.5 ± 0.2 ^A,B^	5.1 ± 0.4 ^A^	17.1 ± 0.3 ^A^	18.9 ± 0.2 ^B^	21.5 ± 0.5 ^C^	21.5 ± 0.3 ^C^	21.3 ± 0.7 ^C^
**Ratio QC4′/QC**	2.2 ± 0.2 ^A^	2.4 ± 0.5 ^A^	2.4 ± 0.1 ^A^	2.1 ± 0.1 ^A^	2.4 ± 0.4 ^A^	0.54 ± 0.01 ^C^	0.60 ± 0.02 ^D^	0.55 ± 0.02 ^C^	0.49 ± 0.01 ^B^	0.43 ± 0.02 ^A^

ND: Not detected. * Total QCs means the sum of QC, QC4′, QC3 and QC3,4′. Different letters indicate statistically significant differences at confidence level of 95%, among the results obtained at each temperature for both total and soluble fraction. ** Total QCE means total quercetin equivalents, calculated according to Equation (1). Different letters indicate statistically significant differences at confidence level of 95%, among the results obtained at each temperature for both total and soluble fraction.

## Supplementary Materials

The following are available online at https://www.mdpi.com/2076-3921/9/12/1233/s1, Figure S1. Example of a HPLC/DAD chromatogram (wave length 280 nm) of a sample obtained from OSW at 145 °C and 5 MPa; Table S1: Comparison between standards’ UV spectrum and unknown peaks’ spectrum found in OSW samples obtained using subcritical water.
