# Supplementary material for: Subcritical Water Extraction of Phenolic Compounds from Onion Skin Wastes (Allium cepa cv. Horcal): Effect of Temperature and Solvent Properties
Source: Antioxidants (Basel). 2020 Dec 4;9(12):1233. doi: 10.3390/antiox9121233 (PMC7762022; doi:10.3390/antiox9121233)
Supplement: Supplementary file 1 [file antioxidants-09-01233-s001.pdf]

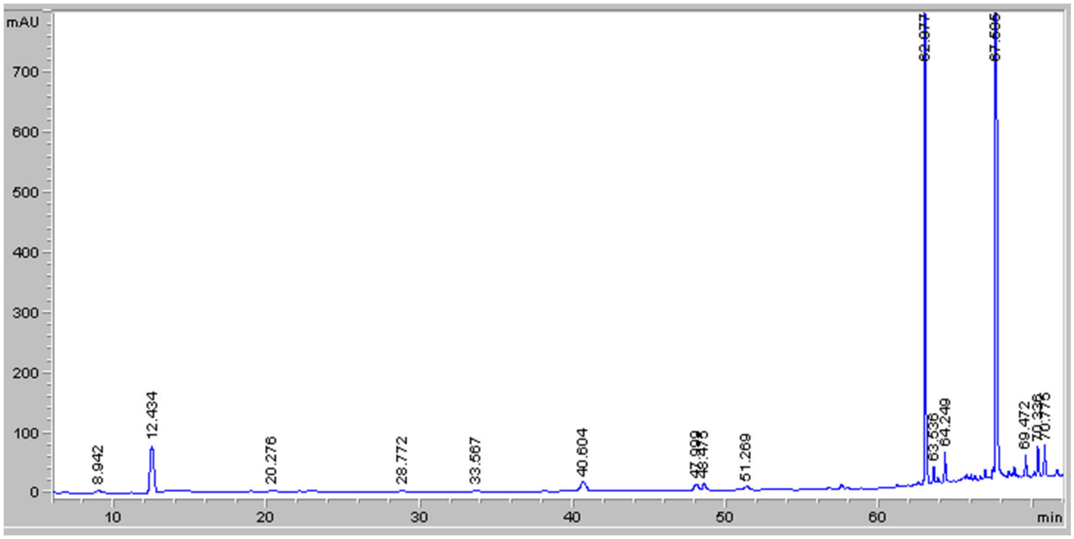

Figure S1. Example of a HPLC/DAD chromatogram (wave length 280 nm) of a sample obtained from OSW at 145 °C and 5 MPa

Table S1. Comparison between standards' UV spectrum and unknown peaks' spectrum found in OSW samples obtained using subcritical water. Red line represent the standards' spectrum and blue line represents the unknown peaks' spectrum

Compound	Retention time (min)	UV spectra	Match (%)
QC	Standard: 67.37<br>Peak: 67.55	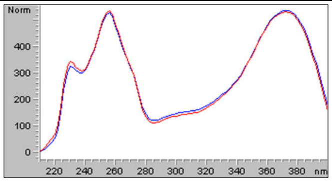	98.8±0.7
QC4′	Standard: 62.8<br>Peak: 62.77	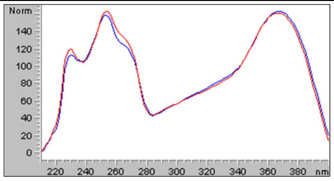	99.2±0.4
QC3,4′	Standard: 47.39<br>Peak: 47.56	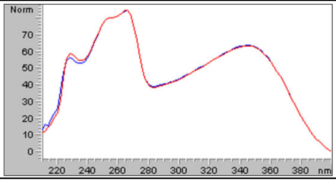	99.7±0.1
QC3	Standard: 56.40<br>Peak: 56.56	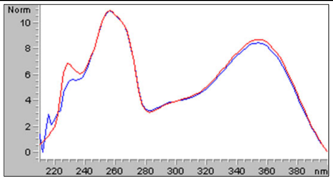	97.4±0.9
Kaempferol	Standard:70.11<br>Peak: 70.34	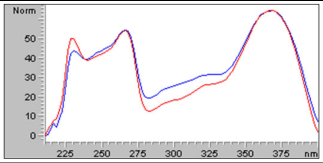	96.2±0.8
Isorhamnetin	Standard: 70.62<br>Peak: 70.77	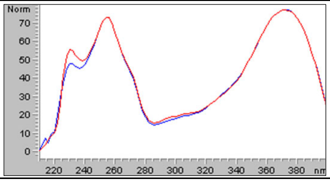	99.3±0.2
Protocatechuic Acid	Standard: 12.56<br>Peak: 12.43	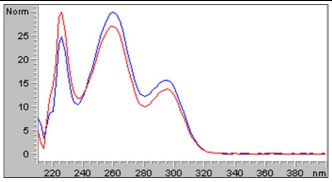	96.2±0.7
